# Nurses’ and Doctors’ Experiences of Transferring Adolescents or Young Adults With Long-Term Health Conditions From Pediatric to Adult Care: A Metasynthesis

**DOI:** 10.1177/23333936231189568

**Published:** 2023-08-07

**Authors:** Liv Fegran, Thomas Westergren, Elisabeth O. C. Hall, Hanne Aagaard, Mette Spliid Ludvigsen

**Affiliations:** 1 University of Agder, Kristiansand, Norway; 2 University of Stavanger, Norway; 3 University of Faroe Islands, Torshavn, Faroe Islands; 4 Aarhus University, Denmark; 5 Lovisenberg diaconal University College, Oslo, Norway; 6 Nord University, Bodø, Norway

**Keywords:** nurse, doctor, adolescent, young adult, transition to adult care, qualitative systematic review, Sykepleier, lege, ungdom, ung voksen, overgang til voksenomsorg, metasyntese, systematisk litteraturstudie

## Abstract

The transfer of adolescents and young adults (AYA) with long-term health conditions from pediatric to adult care is a multidisciplinary enterprise where nurses and doctors play an important role. This review aimed to identify and synthesize evidence from qualitative primary reports on how nurses and doctors experience the transfer of AYA aged 13 to 24 years with long-term health conditions to an adult hospital setting. We systematically searched seven electronic databases for reports published between January 2005 and November 2021 and reporting nurses’ and doctors’ experiences. We meta-summarized data from 13 reports derived from 11 studies published worldwide. Using qualitative content analysis, we metasynthesized nurses’ and doctors’ experiences into the theme “being boosters.” Boosting AYA’s transfer was characterized by supporting AYA’s and their parents’ changing roles, smoothening AYA’s transition from pediatric to adult care, and handling AYA’s encounters with a different care culture.

## Abstrakt

Overføring av ungdom og unge voksne med kroniske sykdommer fra pediatrisk- til voksenomsorg er et tverrfaglig samarbeid der sykepleiere og leger har en viktig rolle. Hensikten med denne metasyntesen var å identifisere og syntetisere data fra kvalitative primærstudier som omhandlet sykepleieres og legers erfaringer med overføring av ungdom og unge voksne (13-24 år) fra pediatrisk- til voksenomsorg. Vi søkte systematisk i sju elektroniske databaser etter studier publisert mellom januar 2005 og november 2021 som omhandlet sykepleieres og legers erfaringer. Vi inkluderte data fra 13 publikasjoner fra hele verden. Data ble analysert ved kvalitativ innholdsanalyse, og vi fant at sykepleieres og legers erfaringer kunne beskrives som å være «hjelpere» i denne overgangen. Overføringen var preget av å støtte de unge og foreldrene når rollene deres ble endret, tilrettelegge overgangen mellom pediatrisk og voksenomsorg, og støtte de unge i møte med en ny omsorgskultur.

## Introduction

Young people’s transition from pediatric to adult care is a multidisciplinary enterprise, and nurses and doctors play an important role in the transfer team to assist young people to become responsible and independent (Coyne & Betz, 2020; Fegran et al., 2014; Mackie et al., 2018). An increasing number of studies concerned only with adolescents’ and young adults’ (AYA’s) experiences of the transfer process have emphasized the importance of a close collaboration between young people and healthcare providers (HCP) from various professions to enable a successful transition from pediatric to adult care (DeSouza et al., 2019; Fegran et al., 2014; Kerr et al., 2017; Sattoe et al., 2017). Research on nurses’ and physicians’ experiences of the transition of AYA appear to be scarce, even if their participation and knowledge both can facilitate and hinder the process. (Lestishock et al., 2018; Meleis et al., 2000). Thus, in this metasynthesis study, we explored the nurses’ and doctors’ experiences regarding the transition of AYAs with long-term health conditions to adult care.

## Background

The number of children and adolescents growing up with long-term conditions is increasing worldwide (Aldiss et al., 2015). Approximately 20% of AYA in the US are affected by special health conditions, and more than a fifth of them need to move to the adult care system and continue to receive support and care (Ghandour et al., 2022).

Structural, clinical, and individual benchmarks have been identified to improve the transfer of AYA with long-term health challenges to adult care (Aldiss et al., 2015; Mora et al., 2019). Continuity of care and interclinic communication appeared to be essential issues during young people’s transition (Aldiss et al., 2016; Campbell et al., 2016; Straub & Tanner, 2018). Ensuring that the patient is not lost to follow-up is crucial for a successful transition (Suris & Akre, 2015). Moreover, the identification of the most appropriate time for transfer is an important issue for the HCPs. In a metasynthesis about young people’s experiences of transfer, Fegran et al. (2014) found that AYA considered readiness and maturity as being more important than biological age and that transfer at the age of 18 to 19 years appeared to be most appropriate. These findings are in accordance with those of Yassaee et al.’s (2019) systematic review about the impact of age on transfer, which concluded that a delayed transfer resulted in improved outcomes and an age of 18 years was appropriate for the transfer.

Previous studies reported that HCPs showed a genuine willingness to ease the transfer between pediatric and adult care for young persons, and several statements, guidelines, and intervention models regarding this topic have been developed and implemented over the last decades (Betz et al., 2014; Bower et al., 2017; Chu et al., 2015; Coyne & Betz, 2020; Foster et al., 2017; Mora et al., 2019; Walter et al., 2018). However, the evidence for these interventions is scarce. Campbell et al. (2016), in their review evaluating the effectiveness of interventions designed to improve the transition of care for AYA from pediatric to adult health services, concluded that the evidence was not sufficient to draw any firm conclusions about the effectiveness of the evaluated interventions. In a review of the impact of transition interventions, Chu et al. (2015) identified that the intervention (defined as attending at least one appointment with an adult) was associated with increased rates of transfer in three of five studies, while no statistically significant effects were noted in the remaining two studies.

Despite the growing concern on nurses’ and doctors’ experiences of handling of AYAs transfer, the transition process is repeatedly described as challenging both in pediatric and adult care and primary care settings (Aldiss et al., 2015; Garvey et al., 2016; W. N. Gray et al., 2015; Scal, 2002; Straub & Tanner, 2018). Nurses’ and doctors’ crucial role in facilitating AYA’s transition has been emphasized (Coyne & Betz, 2020; Fegran et al., 2014). Collaboration between pediatric and adult care teams continues to be worse than expected (McManus et al., 2015) and nurses’ and doctors’ participation and knowledge could both facilitate and hinder the process (Lestishock et al., 2018; Meleis et al., 2000). However, studies concerned only on nurses’ and doctors’ experiences regarding AYA’s transition are limited, and Nehring et al.’s (2015) systematic review of 55 studies claimed this research area to be “uncharted territory” with a lack of evidence.

We conducted a preliminary search for previous systematic reviews on this topic in databases such as the JBI Database of Systematic Reviews and Implementation Reports, Cochrane Database of Systematic Reviews, CINAHL, PubMed, and PROSPERO, and identified no current or underway systematic reviews. Thus, this review aimed to identify and synthesize the best available evidence from qualitative primary reports on the nurses’ and doctors’ experiences regarding the transition of young people with long-term conditions from pediatric to adult hospital care. The objectives were to explore nurses’ and doctors’ experiences of (1) preparing young people for their transfer from pediatric to adult care; (2) continuity of care between pediatric and adult care; and (3) using standardized procedures to facilitate the AYAs transfer.

## Methods

To contribute to a valid synthesis (Ludvigsen et al., 2016), we followed the now to be mentioned six-step research synthesis methodology proposed by Sandelowski and Barroso (2007): (1) conceiving the synthesis; (2) searching and retrieving the literature; (3) appraising reports; (4) classifying the findings; (5) synthesizing findings into metasummaries; and (6) synthesizing qualitative findings into a metasynthesis. The metasynthesis is based on a previously published protocol in the JBI Database of Systematic Reviews and Implementation Reports (Fegran et al., 2016). In the reporting, we adopted the principles underlying the Preferred Reporting Items for Systematic Reviews and Meta-Analyses 2020 guidance and exemplars for reporting systematic reviews for data retrieval in terms of identification, screening, eligibility, and inclusion (Page et al., 2021).

To define our research questions and develop the systematic search strategy, we used the PICO mnemonic for qualitative studies (Cooke et al., 2012). The review population was nurses or doctors with experience(s) of transferring AYA aged 13 to 24 years (adolescent is a person 13–18 years of age, and a young adult is a person between 19 and 24 years of age) with long-term health conditions, regardless of gender, ethnicity, or country of origin. Studies focusing on nurses’ and doctors’ experiences of the transferring AYA residing in residential accommodations or institutionalized care or those with mental conditions/intellectual disabilities were excluded.

Acknowledging that a transition is more than a transfer (Meleis et al., 2000), the phenomenon of interest in this review was the transfer as an event, thus referring to an external move, that is, the change or relocation in relation to a healthcare transfer. The target phenomenon was preparation, implementation, and reflections on transfers (both inter- and intrahospital transfers). In contrast to transfer as an external event, we considered the concept of transition as an internal change process triggered by the external transfer, which is a change that alters an individual’s perception of self and challenges their identity because of several simultaneous changes (Meleis et al., 2000).

The review context was hospital settings, including outpatient clinics. Another inclusion criterion was that the primary study should either describe AYA’s transfer from a pediatric to an adult unit in the same hospital (intrahospital transfer) or from a pediatric hospital unit to an adult hospital clinic (interhospital transfer). The study types considered were those focusing on qualitative data, including, but not limited to, designs such as phenomenology, grounded theory, ethnography, action research, and feminist research.

### Search Strategy

To ensure a thorough search process, an information specialist assisted in designing the systematic searches. A three-step strategy aimed to locate both published and unpublished studies was used (Lockwood et al., 2020). First, we conducted a broad search in the MEDLINE and CINAHL databases using the keywords doctor, nurse, transfer, transition, adolescent, and young adult. Second, we conducted a more extensive search string in collaboration with a librarian. Final searches included all identified keywords and index terms in Embase, MEDLINE and PsycInfo (Ovid), Ovid Nursing, CINAHL (EBSCO*host*), Mednar, and ProQuest for reports published between January 1, 2005, and November 23, 2021. The complete database search strategies are provided in Supplemental File 1.

We uploaded all records to EndNote reference manager version 9.3 (EndNote 20, 2022) and removed duplicates according to Bramer et al. (2016). Furthermore, we uploaded the unique records to Rayyan, which is a web tool designed to help researchers expedite the initial blinded screening of abstracts and titles (Ouzzani et al., 2016). Two reviewers (LF and TW) performed a blinded screening of titles and abstracts. Then, three reviewers (LF, MSL and EH) read the full text of the selected reports and excluded those that did not meet inclusion criteria. Finally, another reviewer (HAA) screened reference lists of all included reports for additional reports.

A substantial challenge during the inclusion process was to identify reports that provided data from our population, nurses and doctors. In many reports, data from the HCPs were grouped, and reporting of the results was not differentiated according to their profession. Because only nurses’ and doctors’ perspectives were to be included, we delineated their views from those of other HCPs, such as social workers, transfer coordinators, patient coordinators, teachers, audiologists, trainers, and researchers. Data were extracted only for nurses and doctors after thorough reading of the full texts.

#### Methodological Quality Appraisal

Two of the reviewers (Y and Z) assessed the methodological quality of the reports using Joanna Briggs Institute’s Critical Appraisal Checklist for critical and interpretive research (Lockwood et al., 2015), and any discrepancies were discussed with the first author (Supplemental File 4).

### Data Analyses

We used Page et al.’s (2021) definition of *a study* as “an investigation, such as a clinical trial, that includes a defined group of participants and one or more interventions and outcomes.” A study might have multiple *reports* defined as “a document (paper or electronic) supplying information about a particular study” (Page et al., 2021 ^p. 3^). After deciding on the 13 reports (11 studies) to be included, we created metasummaries of characteristics of our reports, and developed themes of condensed raw data about nurses’ and doctors’ experiences. Finally, from the metasummaries and the themes, we created a comprehensive metasynthesis of the nurses’ and doctors’ experiences of AYAs transition (Sandelowski & Barroso, 2007).

Conducting metasummaries involves the quantitative aggregation of qualitative findings while searching for patterns and hypotheses. To provide a general picture of the study characteristics, we extracted information about the author, publication year, country of publication, aims, setting (context), type of chronic condition, participants, methodology, analysis, and findings (Table 1).

**Table 1.** Characteristics of Included Studies.

Reference, year, country	Country	Aim	Setting (context)	Children’s chronic condition	Participants included in the metasynthesis	Other participants than nurses and physicians	Methodology and method	Analysis	Findings
Reiss et al. (2005)	USA	Identity practices and factors that make transition for youths and young adults with disabilities and SHCNs successful	Disability and health care needs based on a range of chronic illnesses and disabilities Children’s hospitals, outpatient clinics and treatment programs, community medical centers,	Disabilities and special health care needs	Pediatric ward: 7 MD and 25 nurses	1 psychologist 9 social workers 4 administrators 4 others 44 family members)	Qualitative approach. Focus groups and individual interviews	Content analysis (Huberman and Miles)	Factors that affect transition, Stages of transition, Health care systems
Lundin et al. (2007)	Sweden	Explore how care providers handle the transition process and describe their perception of adolescents’ needs during the process	outpatient clinics	Diabetes	Pediatric and adult ward: 3 physicians 7 nurses	0	Ethnographic approach. Participant observation and semi-structured interview	Grounded theory analysis (Charmaz and Strauss & Corbin)	Core phenomenon: Enabling integration through professional meetings including three main categories and many subcategories (table)
Fair et al. (2010)	USA	To identify best practices recommended by infectious disease medical providers and social workers.	Medical centers and hospitals.	HIV	Pediatric ward: 4 doctors and 5 nurses	10 social workers	Interviews	Qualitative data analysis	Recommended practice for transitioning was promoting medical independence among adolescents; close communication between pediatric and adult providers; and addressing system level concerns, as well as having a separate clinic for adolescents with HIV.
O’Sullivan-Oliveira et al. (2014)	USA	Investigate current state of provider perceptions regarding transition of pediatric patients to adult care	Free-standing, urban, pediatric hospital	NA	Pediatric ward: 7 MD, 8 NP, 4 RN	8 social workers 1 physician assistant	Qualitative approach. Focus groups	Phenomenological thematic analysis Van Manen	Themes: (1) complex patients, provider barriers (2) patient and parents’ resistance (3) provider recognition of importance (4) institutional support resources
Newman et al. (2014)	Australia	Explore how the health and social care clinicians who care for young people with perinatally acquired HIV.	HIV health services	HIV	Pediatric ward: 5 doctors, adult care 4 doctors	3 with nursing or allied health background	Exploratory qualitative study. Individual interviews.	Constant comparison analysis (Thorne)	Themes: (1) anticipating client vulnerabilities (2) challenging professional boundaries (3) navigating two worlds
Philbin, Tanner, Ma, et al., 2017 (CATCH study)	USA	Identify key components of successful transition	4 Adolescent Medicine Trials Network (ATN) clinical sites and 20 adult clinics	HIV	18 physicians 9 nurse practitioners 17 physicians 9 Nurse practitioners 11 physicians 2 Nurse practitioners Pediatric and adult ward: 18 physicians 9 NP	13 Social worker 6 Case manager 7 Linkage to care/ patient coordinator or supervisor 5 Other	Qualitative approach. Semi-structured individual interviews	Constant comparative method (Glaser & Strauss)	Core phenomena: (1) Youth knowing (2) youth taking responsibility (3) youth feeling connection and trust
Philbin, Tanner, Chambers, et al., 2017 (CATCH) Study	USA	Identify key components of transition barriers	14 Adolescent Medicine Trials Network (ATN) clinical sites and 20 adult clinics	HIV		13 Social Worker 5 Case Manager 6 Linkage to care/patient coordinator or supervisor 6 Other	Qualitative approach. Semi-structured individual interviews	Constant comparative method (Glaser & Strauss)	Themes related to barriers on three levels: structural, clinical, individual
(Tanner et al. (2017) (CATCH) Study	USA	Examine HIV-related health care transition approaches and processes from the perspective of adult clinic staff	Adult HIV clinics	HIV		5 Social Workers 3 Case Manager 4 Care linkage/patient coordinator or supervisor 3 Other (pharmacist, family advocate, substance abuse specialist/retention staff)	Qualitative approach. Semi-structured individual interviews	Constant comparative method	Core phenomena: (1) clinic characteristics (2) transition process and evaluations (3) adult clinical culture (4) expectations
Pinzón-Iregui et al. (2017)	Dominican Republic	Characterize transition experiences in the Dominican Republic	A comprehensive care facility	HIV	Not described	7 health care providers in pediatric or adult health care including nurses, physicians, psychologists, and social workers	Qualitative approach. Focus group interviews	Grounded theory and phenomenological approach	Core phenomena: transition process, adherence, stigma and discrimination
Le Roux et al. (2017)	France	Explore how HIV professionals working in adult care perceive and adapt their practices to young people in transition	Pediatric research hospital	HIV	Pediatric ward: 9 medical doctors 3 nurses	1 social worker 3 auxiliary nurses 4 psychologists	Qualitative approach. Semi-structured individual interviews	Thematic analysis	Themes related to: (1) problems encountered during transition (2) elements acting for a smooth transition (3) impact of transition
Wright et al. (2019)	UK	Elicit views of stakeholders about barriers and facilitators of an effective transition process	pediatric and adult liver transplant centers	Liver transplantation	Pediatric ward: 1 doctor and 2 nurses, and adult ward: 1 doctor and 3 nurses	4 allied health professionals	Qualitative approach. Semi-structured individual interviews	Thematic analysis	Themes: (1) non-adherence and psycho-social issues (2) need for better psychological support (3) the role of parents (4) the emotional impact of transition on HCPs
Gabay Gillie and Tarabeih (2020)	Israel	Focus on trust relationships of nurses with YA upon their transition to adult renal care	Tertiary hospitals	Kidney transplantation	Adult care: 11 nurses	0	Qualitative approach. Thematic individual interviews.	Thematic/narrative analysis	Core phenomena: (1) role and communication (2) building a trust relationship
(Bitencourt et al. (2021)	USA	To explore clinical team members’ beliefs regarding the transition from pediatric to adult rheumatology for patients with childhood-onset SLE.	2 pediatric and 4 adult clinical practice sites in a metropolitan area.	Systemic Lupus Erythematosus	Pediatric care: 4 rheumatologists, adult care: 4 rheumatologists Not specified ward: 2 nurses and 1 nurse practitioner	1 social worker 1 psychologist	A constructivist grounded theory approach. Semi-structured in-depth interviews	A constant comparative method	Five themes: (1) achieving balance in family involvement developing resilience and coping strategies (2) role of mental health and emotional Support (3) need for and challenges with health Education (4) importance of peer support (5) social connectedness

For the metasynthesis, we imported all included reports into the computer software (NVivo version 12) for analysis according to Graneheim and Lundman’s (2004) method of qualitative content analysis. We derived target findings from the result chapters of the included reports as quotations, paraphrases, observations, or the primary researchers’ interpretation (Sandelowski & Barroso, 2007). Other findings which were excluded were experiences from HCPs other than nurses and doctors in the transition care team, descriptions of analytical procedures, findings imported from other reports, or discussion of results. Two reviewers independently reviewed these texts to clarify whether the data could provide findings of nurses’ and doctors’ experiences regarding the transfer of AYA to adult care.

The first step in the qualitative content analysis was to create condensed meaning units from raw data, which comprise words, sentences, or paragraphs containing aspects related to each other through their content and context. Next, we grouped these meaning units into categories expressing the manifest content of the text. Based on their name and content, we interpreted the categories and integrated them into themes describing the latent meaning of findings. Finally, we synthesized the themes into a comprehensive whole. In this integrative process, we adapted the notion “discussions about being boosters” as the metasynthesis of nurses’ and doctors’ experiences regarding the transition of young people to adult care. The main analysis was conducted by two of the authors, though all reviewers participated in repeated discussions until consensus was reached. Examples of the analyses from raw data to themes are presented in Table 2.

**Table 2.** Examples of the Analysis From Raw Data to Themes.

Raw data	Condensed meaning unit	Categories	Themes
“Adult providers expected independent, proactive, and adherent patients, especially around missed visits. As one adult physician (X-A) noted: ‘If you’re lucky, you’ll get a reminder the day or two before, an automated machine, and that’s pretty much all the assistance you have.’”(Philbin, Tanner, Chambers et al., 2017)	Expecting engagement from the young people	Shift of role during transition	Supporting AYA’s and their parents’ changing roles
“One pediatric rheumatologist noted that some families develop a “chronic illness mindset” [such that it is] harder for parents to take their hands off and step back.” (Bitencourt et al., 2021)	Harder for parents to let go		
“To develop an autonomous YA, capable of acting responsibly, independently, sexually, and socially. To prevent complications by communicating in a way that fits their needs, so they don’t drop out of care.” (Gabay Gillie & Tarabeih, 2020)	Communicate in a way that makes AYA become independent	Building a relationship with AYA	Smoothening AYA’s transition from pediatric to adult care
“‘Look, they can see anyone’. And I know that we should never think of ourselves as indispensable, and we’re not. [But] you work a lot harder to build relationships with adolescents, so I think, if you’re going to get involved . . . you have to do it with a mind that you’re going to be around for a while.” (Newman et al., 2014)	Prioritize time to care for the young person		
“Interviewees reported that first contact with the adult service, where medical and technical language prevails, where care workers are less patient, available or attentive, where young patients are considered as independent adults, and where the population of the waiting room is very different, can be a rude shock.” (Le Roux et al., 2017)	Culture crash	Understanding different caring cultures	Handling AYAs encounter with a different care culture
“A pediatric medical provider [doctor] said, “And then I think, also, having the pediatric provider and the adult provider in close communication with each other over a transition period is ideal.”” (Fair et al., 2010)	Communication between the two wards		

### Effect Sizes

To provide a more comprehensive understanding of the data, we calculated the magnitude of the extracted findings by describing which reports contributed to each of the three themes (interstudy effect size) and the concentration of findings in each study (intrastudy effect size) as presented in Supplemental File 2 (Onwuegbuzie, 2003). We presented inter- and intrastudy effect sizes to assess the study validity by calculating the magnitude of the extracted findings (Onwuegbuzie, 2003).

## Results

The systematic searches in seven databases identified4,579 records. After removal of duplicates, we screened2,983 records for inclusion by title and abstract, which provided 81 reports to be assessed for eligibility. Of these 81, we excluded 69, leaving 13 reports to be included. The reasons for excluding the 69 reports were as follows: (1) not possible to extract data from nurses/doctors (*n* = 25); (2) not regarding nurses’ and doctors’ experiences (*n* = 15); (3) not primary studies (*n* = 18); (4) not a long-term health condition (*n* = 8); (5) no qualitative data (*n* = 3); or (6) wrong language (*n* = 1) (Supplemental File 3). Through reference list checking of the 12 included reports, one more report was included. Thus, the systematic inclusion process resulted in 13 reports representing 11 study populations (80 nurses and 67 doctors). Three reports were published from the same CHASE study (Philbin, Tanner, Chambers, et al., 2017; Philbin, Tanner, Ma, et al., 2017; Tanner et al., 2017). The search strategy and inclusion process are presented in a flow diagram according to the Preferred Reporting Items for Systematic Reviews and Meta-Analyses guidelines (Page et al., 2021).

Except for the two questions regarding a statement locating the researcher culturally or theoretically (Q6), or reflections about the influence of the researcher on the research and vice versa (Q7), all included reports were assessed to have a sound methodological quality. In line with Sandelowski and Barroso’s (2007) advice, no studies were excluded because of of methodological weaknesses if they provided relevant data for the synthesis. The effect sizes (Supplemental File 2) were calculated to visualize more meaning from the analyzed data, and to verify the presence of pattern or themes (Sandelowski & Barroso, 2003). The inter study effect sizes (findings *within* each report) varied between 33 and 100%, and the intra-study effect sizes (the number of reports providing data for each theme) varied from 62 to 92%. By calculating the effect sizes, we attempted to avoid under- or over-estimating our findings, and assure the quality of our analysis.

### Characteristics of Included Reports

The 13 included reports (Table 1) represented research conducted primarily in North America (*n* = 8); the others were from Europe (*n* = 3), Asia (*n* = 1), and Oceania (*n* = 1). The total number of nurses and doctors was 147 (nurses, *n* = 80; doctors, *n* = 67). In most reports (*n* = 7), the prominent long-term condition was human immunodeficiency virus (HIV) infection. Most participants worked in pediatric wards (nurses, *n* = 47; doctors, *n* = 37), while 14 nurses and nine doctors worked in adult wards. In two of the reports, the authors did not specify the type of ward (nurses, *n* = 19; doctors, *n* = 21). Three of the included reports came from the same sample, which was confirmed by the primary investigator of the CHASE study (Philbin, Tanner, Chambers, et al., 2017; Philbin, Tanner, Ma, et al., 2017; Tanner et al., 2017). Thus, the participants from this study were only counted once. In two of the reports, the authors applied a specific qualitative research design (ethnographic approach and grounded theory), while in 11 reports the authors applied unspecified qualitative research designs. Individual interviews were the most used data collection method (*n* = 8), in addition to focus group interviews (*n* = 3), a combination of focus group and individual interviews (*n* = 1), and individual interviews and observations (*n* = 1). Furthermore, the primary researchers used different methodological approaches with the constant comparative method (*n* = 5) and thematic analysis (*n* = 4) being the most frequently used; the rest being grounded theory analysis (*n* = 2), content analysis (*n* = 1), and qualitative analysis (*n* = 1).

#### Meta-Synthesis Findings

This review of nurses’ and doctors’ experiences and roles in the transition of AYA with long-term conditions to adult care was based on the data from 13 qualitative reports published between 2005 and 2021. An overarching synthesis of our findings was the concept of “being boosters.” We found that nurses and doctors discussed their experiences of what could booster the transfer of AYA from the pediatric to the adult ward. The following three themes formed the basis of our understanding: supporting AYA’s and their parents’ changing roles, smoothening AYA’s transition from pediatric to adult care, and handling AYA’s encounters with a different care culture. These themes are described in the text that follows.

### Supporting AYA’s and Their Parents’ Changing Roles

A prominent finding was that supporting not only AYA’s but also their parents’ changing roles and responsibilities related to the transition/transfer was often crucial. The young people’s role evolved from being constantly supported and taken care of, to gradually assuming full responsibility for their health and future (Newman et al., 2014). However, nurses and doctors feared that adherence to treatment and follow-up by AYA would be reduced during the transition (Gabay Gillie & Tarabeih, 2020; Pinzón-Iregui et al., 2017). For nurses and doctors caring for patients with HIV infection, adherence was a characteristic of a successful transition ( Philbin, Tanner, Ma, et al., 2017). During the transfer, adult nurses and doctors expected AYA to be proactive and independent. Issues such as identifying the type of meetings and strategies that were appropriate for them (Lundin et al., 2007) and strategies to avoid them from dropping out from appointments or follow-ups were important (Philbin, Tanner, Chambers, et al., 2017). Involving young people in their transition was described as crucial to support their journey to independency (Le Roux et al., 2017; Newman et al., 2014). Reduced disclosure with pediatric nurses and doctors before moving on to adult care was described as a way to prevent encountering AYA with a paternalistic approach (Newman et al., 2014). The nurses and doctors expressed a need to discuss these issues with the young people, and the young people’s engagement would facilitate these challenging conversations. Relating behavior and consequences could be difficult for young people; thus, they should be involved in the transition process (Lundin et al., 2007; Newman et al., 2014). Their opinions should be heard, because nurses and doctors assuming what was their best could leave young people irresponsible for their health and future (Gabay Gillie & Tarabeih, 2020; Philbin, Tanner, Ma, et al., 2017).

The young people’s changing roles could easily lead to a modified parental role because the responsibility is gradually transferred from the parents to the young adult in adult care (Wright et al., 2019). Preparing parents before the actual transfer appears to be crucial because parents sometimes had a hard time letting their young adult move on (Fair et al., 2010; Reiss et al., 2005). Both pediatric and adult care nurses and doctors experienced a transition from being caretakers to becoming spectators regarding AYA’s gradually developing independence, which sometimes could create an imbalance in the parent—AYA relationship (Bitencourt et al., 2021). This imbalance could be felt as overprotection. Both parents and professionals could be seen hesitant to give up control over the AYA’s chronic condition (Lundin et al., 2007; Reiss et al., 2005; Wright et al., 2019). Nurses and doctors in pediatric ward described their effort to involve the parents ahead of the transfer to underscore AYA’s need for responsibility and independence (Wright et al., 2019). However, in one of the reports (Bitencourt et al., 2021) they found the opposite imbalance when families, instead of being overprotective, showed a lack of engagement in their young adult’s situation. It was suggested that this lack of engagement could be related to various factors, such as chaotic social situations, lack of family role models, and lack of basic stability. Easing these life circumstances also involved nurses and doctors (Bitencourt et al., 2021).

### Smoothening AYA’s Transition From Pediatric to Adult Care

Smoothening the young people’s transition refers to the nurses’ and doctors’ effort to decrease challenges related to sickness and health. Both pediatric and adult care nurses experienced the need for a gradual transition between the wards (Gabay Gillie & Tarabeih, 2020). Moreover, preparation within a certain time frame was necessary. Sufficient time was required to prepare for the transfer until the formal farewell from the pediatric ward (O’Sullivan-Oliveira et al., 2014). However, the transfer of young people from a familiar pediatric context to an unfamiliar adult care context revealed ambivalent feelings among nurses and doctors. Pediatric doctors appeared to miss a continuing relationship with their patients *after* the transfer, while the doctors in adult care could miss being involved *before* the transfer (Newman et al., 2014). In pediatric care, there was a need to let go, but at the same time a strong need to be assured that the young people will be able to cope with what is anticipated of them after the transfer (Newman et al., 2014; O’Sullivan-Oliveira et al., 2014; Reiss et al., 2005; Wright et al., 2019). Pediatric nurses and doctors expressed a desire to help young people to build a trusting relationship with nurses and doctors in adult care and to be assured that the young people were transferred into safe hands (Le Roux et al., 2017). Approaching the young people with an understanding and positive attitude after their transfer to the adult ward was perceived as important when building these relationships (Gabay Gillie & Tarabeih, 2020). Doctors in adult care expressed the need for good acclimatization to make AYA confident during the transition, and some wanted to build a “bridge over troubled water” (Gabay Gillie & Tarabeih, 2020). Accompanying the young adults to their first appointment in adult care, having a peer navigator, identifying a person in the adult care team to be their special contact, or equipping them with the necessary tools and information could assist them in successfully managing their transition (Gabay Gillie & Tarabeih, 2020; Le Roux et al., 2017; Tanner et al., 2017). Nurses and doctors discussed how an adolescent clinic, which refers to a full clinic for AYA 16 to 24 years old, could be a step in decreasing transfer barriers between the two ward cultures (Fair et al., 2010). Finally, sharing a medical record system was described as a way of facilitating the flow of information between wards and easing the transition for AYA (Philbin, Tanner, Chambers, et al., 2017; Tanner et al., 2017).

The AYA’s transfer timing was experienced as closely related to their maturity and the young people’s ability to be responsible and take care of themselves. Although most young people were developmentally mature enough to make the transfer to adult care at the age of 18 to 21 years, nurses and doctors experienced that the consequences of a complex health situation or vulnerability could postpone the transfer (Philbin, Tanner, Chambers, et al., 2017). If the transfer took place too early, it could damage the prepared foundation for a good transfer because the young people were not ready for the next step, and this could lead to poor health and quality of life (Newman et al., 2014). If nurses and doctors delayed the transfer of AYA from pediatric wards, this could reduce their resources for preparing other adult patients for their transition (O’Sullivan-Oliveira et al., 2014). Moreover, they even experienced how young people might experience transition as a punishment caused by their behavior in pediatric care (O’Sullivan-Oliveira et al., 2014).

### Handling AYA’s Encounter With a Different Care Culture

The transfer from the pediatric to the adult ward was seen as a big leap for both nurses, doctors, and young people. Even if the geographic distance was small, it could be experienced as a “feeling like there’s a moat and a wall and maybe a couple of states and an army between the two wards,” as described by a pediatric ward doctor (Reiss et al., 2005, p. 118). To prepare for the young people’s transitions, the nurses and doctors emphasized the need for the young people to understand the difference between the two care cultures (O’Sullivan-Oliveira et al., 2014). The radical change from the pediatric ward, where nurses had a more psychosocial approach to the adolescents’ illness, to the adult ward, where nurses and doctors were much more disease oriented, was crucial for the young people to approve (Le Roux et al., 2017; Reiss et al., 2005).

Preparing for transfer through initial visits was experienced as a way of starting the transition process, as the adolescents’ experiences of this first consultation could be fundamental for their future (Le Roux et al., 2017; Tanner et al., 2017). However, during these initial visits, some adult care nurses appeared to focus more on operational tasks than on making the young people confident in the new setting (Gabay Gillie & Tarabeih, 2020). Our findings revealed a divergent inconsistency concerning a seeming lack of cooperation and a need for overlap between the two wards.

Nurses and doctors in the pediatric ward expressed concern about how the young ones would cope in a new context without the support system available in the pediatric ward (Wright et al., 2019). Nurses and doctors in adult care expected the young people to adhere to the routines and a more disease-oriented approach in adult care (Le Roux et al., 2017). Having a specific transition team or interclinic relationships, as a step between the pediatric and adult wards, could be good for the patients (Fair et al., 2010; Tanner et al., 2017), but not all nurses and doctors felt the same need (Lundin et al., 2007; O’Sullivan-Oliveira et al., 2014; Philbin, Tanner, Chambers, et al., 2017). Nurses and doctors in the pediatric ward suggested that there was a need for training of nurses and doctors in adult wards in understanding AYA’s maturity and needs during transition (Le Roux et al., 2017; Lundin et al., 2007). Nurses and doctors in adult care perceived joint visits as a way of “bridging” between the different wards (Lundin et al., 2007). However, some nurses and doctors in adult care expressed that this was not necessary and that “we’re at a point of overkill, honestly” because they did not have a desire to meet the patients before transition (Lundin et al., 2007; Philbin, Tanner, Ma, et al., 2017).

Thus, nurses and doctors in pediatric care felt the need to protect and support the young ones during their transfer to the adult wards. They experienced that AYA’s fear of moving to a strange and different environment could affect their willingness to be transferred to the adult wards. Such a situation was especially challenging for adolescents with HIV infection who had been more anonymous and protected as pediatric patients (Pinzón-Iregui et al., 2017). Nurses and doctors in adult care described how some adult patients could appear rather scary to young people. These adult patients could visualize their future living with a chronic condition, and this could delay the transition of AYA (Philbin, Tanner, Chambers, et al., 2017).

## Discussion

Disease management of children with long-term health conditions has improved, leading to gradually more children growing into adulthood and in need of care in adult wards. Despite this, nurses and doctors do not always seem to be prepared for this scenario. This systematic review of 13 qualitative reports published worldwide identified nurses’ and doctors’ experiences of transitioning AYA with long-term health conditions from pediatric to adult care. From the interpreted whole of the condensed raw data to themes, we created the comprehensive metasynthesis of nurses’ and doctors’ experiences of AYA’s transition to the metaphor “being boosters.” To boost refers to the nurses’ and doctors’ experiences of how to support AYA’s individual needs during transition.

Teamworking during an AYA transfer is crucial. Nurses and doctors collaborate with participants from several disciplines such as dieticians, psychologists, social workers, and physiotherapists. However specific evidence about nurses’ and doctors’ experiences concerning transfer of AYA is scarce. The limited research interest in the role of nurses and doctors is remarkable because they hold important roles in any multidisciplinary healthcare transfer (Fegran et al., 2014; Kovacs et al., 2016; Lee et al., 2017; Mackie et al., 2018). In Fegran et al. (2014) AYAs describe their relationship with nurses and doctors during transfer as decisive. Their long-lasting relations starting early in life often developed into more than a professional relationship (Kirk, 2008).

In the context of transitions theory (Meleis, 2010), our review findings provided new perspectives about AYA’s transition. We would argue that nurses and doctors in pediatric and adult wards have divergent opinions about what boosters these patients’ transition to a new culture. In addition to the situational transition triggered by relocation from one care culture (pediatrics) to another rather different care culture (adult medicine), being an adolescent or a young adult with a long-term health condition also constitutes being in a developmental transition. According to Meleis et al. (2000), a developmental transition concerns the formation of a personality characterized by a gradually increasing self-identity and independence. The gradually increasing independence of the young people further challenges their relationship with their parents, who experience being cross-pressured and distressed while promoting a safe and predictable transfer (Ludvigsen et al., 2021). These concurrent transitions (situational and developmental) interact with one another and affect the outcomes as levels of mastery and integrative identities (Meleis, 2010).

Supporting the young people’s handling of different care cultures in pediatric and adult care appeared to be a challenge. Even though we found nurses and doctors to be supporting, smoothening, and acting as boosters, data from nurses and doctors in both pediatric and adult care revealed a variety of experiences and opinions. Nurses and doctors in one care culture could be reluctant to trust those in the other care culture. We found a need among pediatric nurses and doctors to have more confidence in adult care nurses and doctors, while the adult caregivers were more divergent in their opinions of what constituted good care for young people. Some nurses and doctors in adult care described how they used time and effort to smoothen the transfer, while others anticipated that the young people easily adjusted to adult care routines and expectations after the transfer. Our findings elaborate on those of Chu et al. (2015) who found that supporting young people’s arrival in adult care was as important as preparing them for the transfer. The importance of acknowledging not only the need for properly preparing for the transition, but also the young people’s needs upon arrival in the new care culture has also been reinforced by others (Coyne & Betz, 2020).

For AYA, the transfer is more than a relocation; it is a transition filled with complexities. If the arrival is neglected, nurses and doctors experienced that the young people might feel displaced among the elderly patients (Le Roux et al., 2017) or they might disregard checkups with fatal consequences for their health (Pinzón-Iregui et al., 2017).

Furthermore, we found that nurses and doctors experienced another transition challenge, which was the changing of roles during the transfer. All reports except one (Bitencourt et al., 2021) mentioned that nurses and doctors experienced the change of role as a crucial part of the young people’s transition. These findings strongly indicate how role transition is important across diagnoses, ward types, and care cultures. During the transition, young people and their parents develop new roles. Parents must take a step back and the young people a step forward in the process of responsibility acceptance for their own health. In agreement with our findings, McLoughlin et al. (2018), while identifying the central role of parents during transition, expressed the need for a phased approach when preparing the families for the transition to a new care culture. However, we would argue with previous researchers (Colver et al., 2019; Sattoe et al., 2017) that transition care should cover both medical and nonmedical challenges, and that parental participation should be appropriate and set aside for special purposes. Too much parental interference may prevent the young people from developing self-management skills and delay the transition to their new role as independent adults.

Our findings of nurses and doctors’ experiences correspond quite well with those from previous reviews about the experiences of adolescents, young adults (Fegran et al., 2014), and their parents’ regarding transitioning from pediatric to adult care (Ludvigsen et al., 2021). Similar to these two reviews, we identified that our findings of nurses’ and doctors’ experiences comprised the following three topics: relationships, context, and roles. However, our review’s contribution expands previous knowledge by only focusing on nurses’ and doctors’ roles during AYA’s transfer. We found them to be important boosters for a successful transition.

## Limitations

Researchers’ influence on the interpretations of results is crucial to assess validity (Hannes et al., 2010). One strength of this review was that the study inclusion, data analysis, and critical appraisal steps were performed by at least two of the authors. Any discrepancy was discussed among the authors until consensus was reached. This was especially crucial when deciding whether it was possible to extract data from nurses and doctors.

More than half of the included reports were about young people diagnosed with HIV infection. This probably influenced our findings and could make the findings less transferable to other chronic diagnoses. However, the three topics supporting independence, adjustment to different care cultures, and smoothening the transfer process appeared to be evident in the previous reviews (Fegran et al., 2014; Ludvigsen et al., 2021), which increases the general understanding of nurses’ and doctors’ roles during the transfer process.

One limitation of this review was that more participants in the included studies represented pediatric wards (nurses, *n* = 47; doctors, *n* = 37) than adult wards (nurses, *n* = 14; doctors, *n* = 9). This dominant representation of pediatric wards could bias the findings because our findings might showcase adult care primarily from the perspective of pediatric care.

Despite the steadily increasing number of studies published on the topic, eligible studies for this metasynthesis were limited. This could be because descriptions or quotations, specifically from nurses and doctors involved in young adults’ transfer, were scarce. The tendency to address the participants as “healthcare team” or “HCPs,” not distinguishing nurses, doctors, or other professionals from other team members could conceal their experiences. This could further complicate the ability to provide evidence for the specific team members’ role.

## Conclusion

Transferring from pediatric to adult care is a multidisciplinary process. However, highlighting nurses’ and doctors’ experiences during the transition of young people could further our understanding of their contribution during the complex transition. Our synthesized findings of nurses’ and doctors’ experiences of being boosters describe the process of preparing and facilitating the transfer from pediatric to adult care. By making the experiences of these two healthcare groups more evident, our findings provide new insights into their experiences regarding their roles in the professional transfer team, which are crucial both in clinical work and education.

## Supplemental Material

sj-docx-1-gqn-10.1177_23333936231189568 – Supplemental material for Nurses’ and Doctors’ Experiences of Transferring Adolescents or Young Adults With Long-Term Health Conditions From Pediatric to Adult Care: A Metasynthesis Click here for additional data file. Supplemental material, sj-docx-1-gqn-10.1177_23333936231189568 for Nurses’ and Doctors’ Experiences of Transferring Adolescents or Young Adults With Long-Term Health Conditions From Pediatric to Adult Care: A Metasynthesis by Liv Fegran, Thomas Westergren, Elisabeth O. C. Hall, Hanne Aagaard and Mette Spliid Ludvigsen in Global Qualitative Nursing Research

sj-docx-2-gqn-10.1177_23333936231189568 – Supplemental material for Nurses’ and Doctors’ Experiences of Transferring Adolescents or Young Adults With Long-Term Health Conditions From Pediatric to Adult Care: A Metasynthesis Click here for additional data file. Supplemental material, sj-docx-2-gqn-10.1177_23333936231189568 for Nurses’ and Doctors’ Experiences of Transferring Adolescents or Young Adults With Long-Term Health Conditions From Pediatric to Adult Care: A Metasynthesis by Liv Fegran, Thomas Westergren, Elisabeth O. C. Hall, Hanne Aagaard and Mette Spliid Ludvigsen in Global Qualitative Nursing Research

sj-docx-3-gqn-10.1177_23333936231189568 – Supplemental material for Nurses’ and Doctors’ Experiences of Transferring Adolescents or Young Adults With Long-Term Health Conditions From Pediatric to Adult Care: A Metasynthesis Click here for additional data file. Supplemental material, sj-docx-3-gqn-10.1177_23333936231189568 for Nurses’ and Doctors’ Experiences of Transferring Adolescents or Young Adults With Long-Term Health Conditions From Pediatric to Adult Care: A Metasynthesis by Liv Fegran, Thomas Westergren, Elisabeth O. C. Hall, Hanne Aagaard and Mette Spliid Ludvigsen in Global Qualitative Nursing Research

sj-docx-4-gqn-10.1177_23333936231189568 – Supplemental material for Nurses’ and Doctors’ Experiences of Transferring Adolescents or Young Adults With Long-Term Health Conditions From Pediatric to Adult Care: A Metasynthesis Click here for additional data file. Supplemental material, sj-docx-4-gqn-10.1177_23333936231189568 for Nurses’ and Doctors’ Experiences of Transferring Adolescents or Young Adults With Long-Term Health Conditions From Pediatric to Adult Care: A Metasynthesis by Liv Fegran, Thomas Westergren, Elisabeth O. C. Hall, Hanne Aagaard and Mette Spliid Ludvigsen in Global Qualitative Nursing Research
