# Supplementary material for: Nurses’ and Doctors’ Experiences of Transferring Adolescents or Young Adults With Long-Term Health Conditions From Pediatric to Adult Care: A Metasynthesis
Source: Glob Qual Nurs Res. 2023 Aug 7;10:23333936231189568. doi: 10.1177/23333936231189568 (PMC10408318; doi:10.1177/23333936231189568)
Supplement: sj-docx-1-gqn-10.1177_23333936231189568 – Supplemental material for Nurses’ and Doctors’ Experiences of Transferring Adolescents or Young Adults With Long-Term Health Conditions From Pediatric to Adult Care: A Metasynthesis [file sj-docx-1-gqn-10.1177_23333936231189568.docx]

**Complete literature search**

**Ovid**

Advanced search mode:

- hand-off = hand off
- health personnel* = "health personnel*"

Adjacency in Ovid

- The ADJ operators finds two terms next to each other in the specified order.
- The ADJ1 operators finds two terms next to each other in any order.
- The ADJ2 operator finds terms in any order and with one word (or none) between them.
- The ADJ3 operator finds terms in any order with two words (or fewer) between them.
- *The ADJ4 operator finds terms in any order and with three words (or fewer) between them, and so on*

Field codes

- .tw = .ti,ab words from title or abstract, word indexed
- .hw = word from subject headings, word indexed
- / = exact Subject heading, phrase indexed
- Exp / = including narrowing subject headings

**OVID MEDLINE. Date. 23.11.2021**

**Database: Ovid MEDLINE(R) ALL <1946 to November 22, 2021>** Search Strategy: [Link](https://ovidsp.ovid.com/ovidweb.cgi?T=JS&NEWS=N&PAGE=main&SHAREDSEARCHID=7VWZTpvfcpLgnDT82lIFmnuFasgw02jvXZ8yGjsj8tAfsW7RqOLmujhRgnBMmiU3p)

--------------------------------------------------------------------------------

1 exp Attitude of Health Personnel/ (165781)

2 ((professional* or nurse* or doctor* or physician* or provider* or staff* or health personnel* or clinican*) adj7 (experience* or comprehension* or attitude* or emotion* or view* or opinion* or perception* or belie* or feeling* or know* or understand* or adaptation* or perspectiv*)).tw. (193834)

3 1 or 2 (320139)

4 ((transition* or transfer* or discharge* or hand-over* or handoff* or handover* or hand-off* or moving) adj7 (adolescen* or young* or youth* or teenage* or adult* or paediatric* or pediatric*)).tw. (23288)

5 Transition to Adult Care/ (1786)

6 Transitional Care/ or Continuity of Patient Care/ (21110)

7 Young Adult/ or Adolescent/ or child/ (3416940)

8 6 and 7 (3566)

9 4 or 5 or 8 (26425)

10 3 and 9 (1252)

**11 limit 10 to yr="2005 -Current" (1076)**

**OVID EMBASE. Date 23.11.2021**

- patient care used for: care, continuity of - continuity of care - continuity of patient care = too broad subject, not used in the search strategy
- EMBASE also includes MEDLINE references – and Conference abstracts

**Database: Embase <1980 to 2021 Week 46> Search Strategy:** [link](https://ovidsp.ovid.com/ovidweb.cgi?T=JS&CSC=y&D=emez&PAGE=main&NEWS=n&ID=&PASSWORD=&SEARCH=exp+health+personnel+attitude/%0A((professional*+or+nurse*+or+doctor*+or+physician*+or+provider*+or+staff*+or+health+personnel*+or+clinican*)+adj7+(experience*+or+comprehension*+or+attitude*+or+emotion*+or+view*+or+opinion*+or+perception*+or+belie*+or+feeling*+or+know*+or+understand*+or+adaptation*+or+perspectiv*)).tw.%0A1+or+2%0A((transition*+or+transfer*+or+discharge*+or+hand-over*+or+handoff*+or+handover*+or+hand-off*+or+moving)+adj7+(adolescen*+or+young*+or+youth*+or+teenage*+or+adult*+or+paediatric*+or+pediatric*)).tw.%0ATransition+to+Adult+Care/%0ATransitional+Care/%0AYoung+Adult/+or+Adolescent/+or+child/%0A6+and+7%0A4+or+5+or+8%0A3+and+9%0Alimit+10+to+yr=%222005+-Current%22)

--------------------------------------------------------------------------------

1 exp health personnel attitude/ (190578)

2 ((professional* or nurse* or doctor* or physician* or provider* or staff* or health personnel* or clinican*) adj7 (experience* or comprehension* or attitude* or emotion* or view* or opinion* or perception* or belie* or feeling* or know* or understand* or adaptation* or perspectiv*)).tw. (249538)

3 1 or 2 (401277)

4 ((transition* or transfer* or discharge* or hand-over* or handoff* or handover* or hand-off* or moving) adj7 (adolescen* or young* or youth* or teenage* or adult* or paediatric* or pediatric*)).tw. (34074)

5 Transition to Adult Care/ (2341)

6 Transitional Care/ (3978)

7 Young Adult/ or Adolescent/ or child/ (2901178)

8 6 and 7 (817)

9 4 or 5 or 8 (34799)

10 3 and 9 (1626)

**11 limit 10 to yr="2005 -Current" (1509)**

**Ovid APA PsycInfo. Date: 23.11.2021**

- Continuum of Care used for Continuity of Care
- No subject headings for age groups, use limit age
- .id = key concept field, word indexed
- .cc = classification code^[[1]](#footnote-1)^

**Database: APA PsycInfo <1806 to November Week 3 2021> Search Strategy**: [link](https://ovidsp.ovid.com/ovidweb.cgi?T=JS&NEWS=N&PAGE=main&SHAREDSEARCHID=3ul5NtntMcdISGYdGMcmmsaCIrX4Y6JzWCczCY3EOaD6C7vIYHQ2tqGKYvQZktMHW)

--------------------------------------------------------------------------------

1 Health Personnel Attitudes/ or "Professional Personnel Attitudes & Characteristics ".cc. (48857)

2 ((professional* or nurse* or doctor* or physician* or provider* or staff* or health personnel* or clinican*) adj7 (experience* or comprehension* or attitude* or emotion* or view* or opinion* or perception* or belie* or feeling* or know* or understand* or adaptation* or perspectiv*)).tw. (108753)

3 1 or 2 (138785)

4 ((transition* or transfer* or discharge* or hand-over* or handoff* or handover* or hand-off* or moving) adj7 (adolescen* or young* or youth* or teenage* or adult* or paediatric* or pediatric*)).tw. (17485)

5 Continuum of Care/ or Client Transfer/ or transition*.id. (22340)

6 limit 5 to (100 childhood <birth to age 12 yrs> or 200 adolescence <age 13 to 17 yrs>) (6196)

7 4 or 6 (20991)

**8 3 and 7 (739)**

**CINAHL (EBSCO*host*). Date: 23.11.2021**

- Information about the database <http://support.ebsco.com/help/?int=ehost&lang=en&feature_id=Databases&TOC_ID=Always&SI=0&BU=0&GU=1&PS=0&ver=live&dbs=cin20jnh,cin20>
- The default fields for unqualified searches consist of the following: Title, Abstract and Subject headings.
- MH = exact subject headings
- MW = words from subject headings
- TI = words from title
- AB = words from abstract
- N# proximity^[[2]](#footnote-2)^

| **#** | **Query** | **Limiters/Expanders** | **Results** |
| --- | --- | --- | --- |
| S1 | (MH "Attitude of Health Personnel+") | Search modes - Boolean/Phrase | 109,738 |
| S2 | AB ((professional* or nurse* or doctor* or physician* or provider* or staff* or "health personnel*" or clinican*) N6 (experience* or comprehension* or attitude* or emotion* or view* or opinion* or perception* or belie* or feeling* or know* or understand* or adaptation* or perspectiv*)) | Search modes - Boolean/Phrase | 131,281 |
| S3 | TI ((professional* or nurse* or doctor* or physician* or provider* or staff* or "health personnel*" or clinican*) N6 (experience* or comprehension* or attitude* or emotion* or view* or opinion* or perception* or belie* or feeling* or know* or understand* or adaptation* or perspectiv*)) | Search modes - Boolean/Phrase | 43,357 |
| S4 | S1 OR S2 OR S3 | Search modes - Boolean/Phrase | 227,440 |
| S5 | MW (adolescen* OR young* OR child*) AND MH ("Transitional Care" OR "Transitional Programs" OR "Health Transition" OR "Continuity of Patient Care") | Search modes - Boolean/Phrase | 4,985 |
| S6 | TI ((transition* or transfer* or discharge* or "hand over*" or handoff* or handover* or "hand off*" or moving) N6 (adolescen* or young* or youth* or teenage* or adult* or paediatric* or pediatric*)) | Search modes - Boolean/Phrase | 4,656 |
| S7 | AB ((transition* or transfer* or discharge* or "hand over*" or handoff* or handover* or "hand off*" or moving) N6 (adolescen* or young* or youth* or teenage* or adult* or paediatric* or pediatric*)) | Search modes - Boolean/Phrase | 9,617 |
| S8 | S5 OR S6 OR S7 | Search modes - Boolean/Phrase | 15,203 |
| S9 | S4 AND S8 | Search modes - Boolean/Phrase | 1,117 |
| S10 | S4 AND S8 | **Limiters - Published Date: 20050101-** Search modes - Boolean/Phrase | **1,026**  [link](https://search.ebscohost.com/login.aspx?direct=true&db=cin20&bquery=(((MH+%26quot%3bAttitude+of+Health+Personnel%2b%26quot%3b))+OR+(AB+((professional*+OR+nurse*+OR+doctor*+OR+physician*+OR+provider*+OR+staff*+OR+%26quot%3bhealth+personnel*%26quot%3b+OR+clinican*)+N6+(experience*+OR+comprehension*+OR+attitude*+OR+emotion*+OR+view*+OR+opinion*+OR+perception*+OR+belie*+OR+feeling*+OR+know*+OR+understand*+OR+adaptation*+OR+perspectiv*)))+OR+(TI+((professional*+OR+nurse*+OR+doctor*+OR+physician*+OR+provider*+OR+staff*+OR+%26quot%3bhealth+personnel*%26quot%3b+OR+clinican*)+N6+(experience*+OR+comprehension*+OR+attitude*+OR+emotion*+OR+view*+OR+opinion*+OR+perception*+OR+belie*+OR+feeling*+OR+know*+OR+understand*+OR+adaptation*+OR+perspectiv*))))+AND+((MW+(adolescen*+OR+young*+OR+child*)+AND+MH+(%26quot%3bTransitional+Care%26quot%3b+OR+%26quot%3bTransitional+Programs%26quot%3b+OR+%26quot%3bHealth+Transition%26quot%3b+OR+%26quot%3bContinuity+of+Patient+Care%26quot%3b))+OR+(TI+((transition*+OR+transfer*+OR+discharge*+OR+%26quot%3bhand+over*%26quot%3b+OR+handoff*+OR+handover*+OR+%26quot%3bhand+off*%26quot%3b+OR+moving)+N6+(adolescen*+OR+young*+OR+youth*+OR+teenage*+OR+adult*+OR+paediatric*+OR+pediatric*)))+OR+(AB+((transition*+OR+transfer*+OR+discharge*+OR+%26quot%3bhand+over*%26quot%3b+OR+handoff*+OR+handover*+OR+%26quot%3bhand+off*%26quot%3b+OR+moving)+N6+(adolescen*+OR+young*+OR+youth*+OR+teenage*+OR+adult*+OR+paediatric*+OR+pediatric*))))&cli0=DT1&clv0=200501-000001&type=1&searchMode=Standard&site=ehost-live) (access is needed) |

Dissertations: **Nursing & Allied Health Database (ProQuest) & Healthcare Administration Database (ProQuest). Date: 23.11.2021**

- Database from ProQuest that includes dissertations that UiA has access to
- APA PsycINFO and CINAHL also includes dissertation
- TI (document title)
- AB (abstract)

| Query: Command line | result |
| --- | --- |
| ti(((professional* OR nurse* OR doctor* OR physician* OR provider* OR staff* OR health personnel* OR clinican*) AND (experience* OR comprehension* OR attitude* OR emotion* OR view* OR opinion* OR perception* OR belie* OR feeling* OR know* OR understand* OR adaptation* OR perspectiv*) AND (transition* OR transfer* OR discharge* OR hand-over* OR handoff* OR handover* OR hand-off* OR moving) AND (adolescen* OR young* OR youth* OR teenage* OR adult* OR paediatric* OR pediatric*))) OR ab(((professional* OR nurse* OR doctor* OR physician* OR provider* OR staff* OR health personnel* OR clinican*) AND (experience* OR comprehension* OR attitude* OR emotion* OR view* OR opinion* OR perception* OR belie* OR feeling* OR know* OR understand* OR adaptation* OR perspectiv*) AND (transition* OR transfer* OR discharge* OR hand-over* OR handoff* OR handover* OR hand-off* OR moving) AND (adolescen* OR young* OR youth* OR teenage* OR adult* OR paediatric* OR pediatric*)))  **Limit: 2005-2022**  **Source type: dissertation & thesis** | **229** |

**Result 23.11.2021**

| MEDLINE (Ovid) | 1076 |
| --- | --- |
| EMBASE (Ovid) | 1509 |
| APA PsycInfo (Ovid) | 739 |
| CINAHL (EBSCO*host*) | 1026 |
| Nursing & Allied Health Database (ProQuest) & Healthcare Administration Database (ProQuest). Date (limit Dissertation) | 229 |
| **Total** | **4579** |
|  |  |
| ***Duplicates removed through EndNote X9.3.3.*** | ***1596*** |
| **Unique** | **2983** |

Compared with already screened titles and abstracts from sept 2020, new more possible relevant hits for screening: **407**

1. <https://www.apa.org/pubs/databases/training/class-codes> 3430.cc = "Professional Personnel Attitudes & Characteristics".cc. [↑](#footnote-ref-1)
2. *Proximity searching is a way to search for two or more words that occur within a certain number of words from each other. The proximity operators are composed of a letter (N or W) and a number (to specify the number of words). The number cannot exceed 255. The proximity operator is placed between the words that are to be searched, as follows: Near Operator (N): N5 finds the words if they are a maximum of five words apart from one another, regardless of the order in which they appear. For example, type tax N5 reform to find results that have a maximum of five words between the beginning and ending terms, that would match tax reform as well as tax that has been submitted for reform. Within Operator (W): W8 finds the words if they are within eight words of one another, in the order in which you entered them. For example, type tax W8 reform to find results that would match tax reform but would not match reform of income tax* [↑](#footnote-ref-2)
