# Supplementary material for: Nurses’ and Doctors’ Experiences of Transferring Adolescents or Young Adults With Long-Term Health Conditions From Pediatric to Adult Care: A Metasynthesis
Source: Glob Qual Nurs Res. 2023 Aug 7;10:23333936231189568. doi: 10.1177/23333936231189568 (PMC10408318; doi:10.1177/23333936231189568)
Supplement: sj-docx-2-gqn-10.1177_23333936231189568 – Supplemental material for Nurses’ and Doctors’ Experiences of Transferring Adolescents or Young Adults With Long-Term Health Conditions From Pediatric to Adult Care: A Metasynthesis [file sj-docx-2-gqn-10.1177_23333936231189568.docx]

# **Supplementary file 2. Inter and intra-study effect sizes**

|  | **Diagnosis** | **Supporting parents’ and young peoples’ changing roles** | **Smoothening the young peoples’ transition from pediatric to adult care** | **Handling the young peoples’ encounter with different caring cultures** | **Inter study effect sizes** |
| --- | --- | --- | --- | --- | --- |
| **A. Tanner et al., 2017** | **HIV** |  | **x** |  | **33%** |
| **Bitencourt et al., 2021** |  | **x** |  |  | **33%** |
| **Newman et al., 2014** | **H**IV | **x** | **x** |  | **67%** |
| **Philbin, Tanner, Ma, et al., 2017** | **H**IV | **x** | **x** |  | **67%** |
| **Philbin, Tanner, Chambers, et al., 2017** | **HIV** | **x** |  | **x** | **67%** |
| **Pinzón-Iregui et al., 2017** | **HIV** | **x** | **x** |  | **67%** |
| **Fair et al., 2010** | **HIV** | **x** |  | **x** | **67%** |
| **Le Roux et al., 2017** | **HIV** | **x** | **x** | **x** | **100%** |
| **Gabay & Tarabeih, 2020** |  | **x** | **x** | **x** | **100%** |
| **Lundin et al., 2007a** |  | **x** | **x** | **x** | **100%** |
| **O'Sullivan-Oliveira et al., 2014** |  | **x** | **x** | **x** | **100%** |
| **Reiss et al., 2005** |  | **x** | **x** | **x** | **100%** |
| **Wright et al., 2019** |  | **x** | **x** | **x** | **100%** |
| **Intra study effect sizes** |  | **92%** | **77%** | **62%** |  |
