# Supplementary material for: Nurses’ and Doctors’ Experiences of Transferring Adolescents or Young Adults With Long-Term Health Conditions From Pediatric to Adult Care: A Metasynthesis
Source: Glob Qual Nurs Res. 2023 Aug 7;10:23333936231189568. doi: 10.1177/23333936231189568 (PMC10408318; doi:10.1177/23333936231189568)
Supplement: sj-docx-3-gqn-10.1177_23333936231189568 – Supplemental material for Nurses’ and Doctors’ Experiences of Transferring Adolescents or Young Adults With Long-Term Health Conditions From Pediatric to Adult Care: A Metasynthesis [file sj-docx-3-gqn-10.1177_23333936231189568.docx]

| Language (n=1) | Becher (2021) |
| --- | --- |
| Not possible to extract data from physicians or nurses (*n =* 24) | Aldiss (2016), Amajjar (2021), Bemrich-Stolz (2015), Bemrich-Stolz (2011), Coyne (2019), Fair (2012), Gilliam (2009), Gray (2015), Hald (2019), Ledford (2015), Li (2021), Lindsay (2017), McCann (2014), McCann (2015), McLoughlin (2018), Nguyen (2016), Olds (2012), Ostlie (2007), Philbin (2017), Sadak (2017), Sangha (2018), Stollon (2015), vanStaa (2011),  Tsang (2021) |
| Not about HCP’s experiences of AYA transfer from pediatric to adult ward (*n =* 15) | Abrams (2008), Bradley (2018), Brez (2009), Fair (2011), Fulton (2019), Gray (2021), Lindsay (2016), Morsa (2020), Nakhla (2017), Newman (2016), Price (2010), Sattoe (2020), Schoenborn (2011), Schraeder (2020), Williams (2021) |
| Not a somatic condition *n =* 8 | Dimitropoulos (2012), Dimitropoulos (2013), Dover (2009), Dunsmuir (2019), Forrester (2011), Leung (2020), Leung (2019), Wright (2016) |
| Not qualitative primary study *n =* 18 | Ancell (2019), Barach (2012), Boyer (2020), Daley (2020), Gleeson (2014), Imbesi (2012), Lampe (2019), Lefkowitz (2011), Lestishock (2018), Melgar (2005), Price (2010), Prufe (2017), Soubra (2014), Stehling (2021), Tsang (2018), Warwick (2017), Watson (2012), Zammit (2017) |
| No qualitative data *n =* 3 | Garvey (2017), Lampe (2019), Walsh (2018) |

**Supplementary file 3. Reason for exclusion after full text reading (n = 69)**
