# Supplementary material for: Nurses’ and Doctors’ Experiences of Transferring Adolescents or Young Adults With Long-Term Health Conditions From Pediatric to Adult Care: A Metasynthesis
Source: Glob Qual Nurs Res. 2023 Aug 7;10:23333936231189568. doi: 10.1177/23333936231189568 (PMC10408318; doi:10.1177/23333936231189568)
Supplement: sj-docx-4-gqn-10.1177_23333936231189568 – Supplemental material for Nurses’ and Doctors’ Experiences of Transferring Adolescents or Young Adults With Long-Term Health Conditions From Pediatric to Adult Care: A Metasynthesis [file sj-docx-4-gqn-10.1177_23333936231189568.docx]

**Supplementary file 4: Critical appraisal results of eligible studies**

| **Author** | **Q1** | **Q2** | **Q3** | **Q4** | **Q5** | **Q6** | **Q7** | **Q8** | **Q9** | **Q10** | **% Yes** |
| --- | --- | --- | --- | --- | --- | --- | --- | --- | --- | --- | --- |
| **(Bitencourt et al., 2021)** | **Yes** | **Yes** | **Yes** | **Yes** | **Yes** | **Yes** | **No** | **Yes** | **Yes** | **Yes** | **90** |
| **(Gabay & Tarabeih, 2020)** | **Yes** | **Yes** | **Yes** | **Yes** | **Yes** | **Yes** | **No** | **Yes** | **Yes** | **U** | **80** |
| **(Lundin et al., 2007)** | **Yes** | **Yes** | **Yes** | **Yes** | **Yes** | **Yes** | **No** | **Yes** | **Yes** | **Yes** | **90** |
| **(Newman et al., 2014)** | **Yes** | **Yes** | **Yes** | **Yes** | **Yes** | **Yes** | **No** | **Yes** | **Yes** | **Yes** | **90** |
| **(O'Sullivan-Oliveira et al., 2014)** | **Yes** | **Yes** | **Yes** | **Yes** | **Yes** | **No** | **No** | **Yes** | **Yes** | **Yes** | **80** |
| **(Philbin, Tanner, Ma, et al., 2017)** | **Yes** | **Yes** | **Yes** | **Yes** | **Yes** | **No** | **No** | **Yes** | **Yes** | **Yes** | **80** |
| **(Philbin, Tanner, Chambers, et al., 2017)** | **Yes** | **Yes** | **Yes** | **Yes** | **Yes** | **No** | **No** | **Yes** | **Yes** | **Yes** | **80** |
| **(Pinzón-Iregui et al., 2017)** | **Yes** | **Yes** | **Yes** | **Yes** | **Yes** | **Yes** | **No** | **Yes** | **Yes** | **Yes** | **90** |
| **(Reiss et al., 2005)** | **Yes** | **Yes** | **Yes** | **Yes** | **Yes** | **No** | **No** | **Yes** | **Yes** | **Yes** | **80** |
| **(Le Roux et al., 2017)** | **Yes** | **Yes** | **Yes** | **Yes** | **Yes** | **Yes** | **No** | **Yes** | **Yes** | **U** | **80** |
| **(Tanner et al., 2017)** | **Yes** | **Yes** | **Yes** | **Yes** | **Yes** | **No** | **No** | **Yes** | **Yes** | **Yes** | **80** |
| **(Wright et al., 2019)** | **Yes** | **Yes** | **Yes** | **Yes** | **Yes** | **Yes** | **No** | **Yes** | **Yes** | **U** | **80** |
| **(Fair et al., 2010)** | **Yes** | **Yes** | **Yes** | **Yes** | **Yes** | **No** | **No** | **Yes** | **Yes** | **Yes** | **80** |
| **% Yes** | **100** | **100** | **100** | **100** | **100** | **46** | **0** | **100** | **100** | **78** |  |

Q1. Congruity between stated philosophical perspective and research methodology? Q2. Congruity between the research methodology and the research question or objective? Q3. Congruity between research methodology and methods used to collect data? Q4. Congruity between the research methodology and the analysis of data? Q5. Congruity between the research methodology and the interpretation of results? Q6. A statement locating the researcher culturally or theoretically? Q7. Influence of the researcher on the research, and vice versa? Q8. Are participants and their voices adequately represented? Q9. Research ethics according to current criteria, ethical approval? Q10. Do the conclusions drawn report flow from the analysis, or interpretation, of the data?
